# An Estimation of Radiobiological Parameters for Head-and-Neck Cancer Cells and the Clinical Implications

**DOI:** 10.3390/cancers4020566

**Published:** 2012-06-15

**Authors:** X. Sharon Qi, Qiuhui Yang, Steve P. Lee, X. Allen Li, Dian Wang

**Affiliations:** 1 Department of Radiation Oncology, University of California Los Angeles, 200 ULCA Medical Plaza, Los Angeles, CA 90024, USA; 2 Department of Radiation Oncology, Medical College of Wisconsin, 8701 Watertown Plank Road, Milwaukee, WI 53226, USA

**Keywords:** head-and-neck cancer, linear-quadratic model, sublethal damage repair halftime, fraction dose delivery time, intensity modulated radiation therapy

## Abstract

*In vitro* survival measurements using two human head-and-neck cancer (HNC) cell lines were performed. The specially designed split-dose surviving fraction was obtained and fitted to the linear-quadratic formalism. The repair halftime (Tr), the potential doubling time (T_d_), α/β and radiosensitivity α, were estimated. Other radiobiological models: EUD, BED, TCP, *etc*., were used to examine the potential treatment effectiveness of different IMRT techniques. Our data indicated the repair halftime of ~17 min based on two HNC cell lines. The combined α/β, α and T_d_ are α/β = 8.1 ± 4.1 Gy, α = 0.22 ± 0.08 Gy^−1^, T_d_ = 4.0 ± 1.8 day, respectively. The prolonged IMRT dose delivery for entire HNC treatment course could possibly result in the loss of biological effectiveness, *i.e*., the target EUDs decreased by 11% with fraction dose delivery time varying from 5 to 30 min. We determined the sublethal damage repair halftime and other radiobiological parameters for HNC cells, and to evaluate treatment effectiveness of the prolonged dose delivery times associated with different IMRT techniques. The estimated repair halftime for HNC is relatively short and may be comparable to the step-and-shoot IMRT fraction dose delivery time. The effectiveness of IMRT treatment may be improved by reducing the fraction delivery time for HNC treatment.

## 1. Introduction

Intensity modulated radiation therapy (IMRT) is becoming the standard technique to treat head-and-neck cancer (HNC) in radiation treatment, since IMRT is capable of delivering highly conformal doses to the target volume while sparing normal structures. However, because of the increasing complexity of the IMRT plan, it generally takes longer time to deliver the same amount of dose as compared to 3D conformal radiotherapy (3DCRT). It is known that the extended IMRT delivery time may reduce treatment effectiveness for cancer cells, specifically for the cancer cells with short repair halftime.

The prolonged IMRT dose delivery for HNC treatment could possibly result in the loss of biological effectiveness. Nowadays, there are various IMRT dose delivery techniques available, such as step-and-shot IMRT, dynamic IMRT, helical Tomotherapy (HT) and recently available volumetric modulated arc therapy (VMAT), *etc*. Among all, the typical dose delivery time may vary in a large range given similar plan quality. For example, a Siemens Primus step-and-shoot accelerator, the estimated fraction dose delivery time can be determined primarily by the number of segments and the total number of MUs per fraction [1]. Given a standard fraction size of 2.0 Gy, the dose delivery time may be as long as 20–30 min depending on the total number of segments; while, the dynamic IMRT delivery time is generally believed to be 1/3 to 1/2 of the static method [2]. For VMAT, the dose delivery is supposedly to be much faster than the step-and-shoot IMRT.

The linear quadratic (LQ) formalism is widely used to analyze both *in vitro* cell survival data [3,4,5,6] and clinical dose response data. Furthermore, the LQ model is also useful to design new radiotherapy treatment regimens *in vivo*, and to study other radiobiological endpoints. The LQ model considers the effects of both lethal damage (double-strand break) and sub-lethal damage by radiation. The dose rate and the cell proliferation effects during radiation treatment have been taken into account in the generalized LQ model [7,8,9]. Parallel analysis of *in vitro* and *in vivo* provides insights in the relationship between clinical response and intrinsic cellular level tumor radiosensitivity. More importantly, the cell survival studies, based on the LQ model, are particularly helpful to provide complementary information that is otherwise not available from *in vivo*.

In this paper, *in vitro* cell survival measurement was performed to measure the repair halftimes, as well as T_d_ and α/β ratios for HNC cell lines. Two cell lines were irradiated by a series of single/split dose regimens using a 6 MV photon beam. The obtained survival data were then analyzed and fitted to the LQ model. A plausible set of radiobiological parameters for HNC, such as sublethal damage repair halftime (T_r_), α and α/β *etc.*, was derived from the *in vitro* cell survival data. The estimated parameter set from *in vitro*, was compared to clinical data based on RTOG 9003 clinical trial [10]. The biological impacts due to the prolonged IMRT fraction dose delivery time and overall treatment duration were carefully calculated using the newly derived parameter set. The radiobiological models, such as biological equivalent dose (BED), equivalent uniform dose (EUD), and tumor control probability (TCP) were used to measure the treatment effectiveness of the prolonged dose delivery time associated with different IMRT techniques. The radiation sensitivity parameters of the LQ parameters, such as α/β, α, T_r_, the tumor doubling time (T_d_) and the variation of lag-off time (T_k_) were also studied.

## 2. Experimental Section

### 2.1. *In Vitro* Experiment

A specially-designed split-dose *in vitro* experiment was performed in this work. Two human HNC cell lines (KB and UMSCC-1) were cultured at 37 °C, in an atmosphere of 5% CO_2_ and 95% air with a complete DMEM growth medium, supplemented with 10% FBS (GIBCO-Invitrogen, Carlsbad, CA, USA). KB cells were derived from a primary epidermal carcinoma of the mouth, and obtained from the American Type Culture Collection (Manassas, VA, USA). UMSCC-1 cells were derived from a primary squamous cell carcinoma of the retromolar trigone, and kindly provided by Thomas Carey (University of Michigan, Ann Arbor, MI, USA). The cells were irradiated with 8 Gy fractions, split in different intervals from 0 to 6 h using a 6 MV photon beam generated by a Siemens Primus accelerator at our department.

#### 2.1.1. Sublethal Repair Halftime Study

UMSCC-1 and KB cells from a stock culture were prepared into a single cell suspension by trypsinization to count cell density. In 100-mm Petri dishes, three thousand cells were seeded and allowed to grow for 24 h. Cells were then irradiated with 8 Gy fractions, split in different intervals: 0, 15 min, 30 min, 45 min, 1 h, 2 h, 4 h and 6 h at our department. A water-equivalent plate of 5 cm thick solid water was placed on the bottom of the flask to ensure the full backscatter condition. Another 1.5 cm thick solid water was placed on the top of the flask to serve as a built-up material for the 6 MV beam. The attached cells were in the bottom of P100 flask at a water-equivalent depth of 5 mm, since they were covered by a 15-mL medium. Therefore, the attached cells were at the depth of dose maximum for the 6-MV (1.6 cm). Four 100 mm Petri dishes were irradiated each time using a 20 × 20 cm radiation field. *In vivo* diode dose radiation measurements were performed to ensure the doses measured matched with the intended doses within 2%. After irradiation, the cells were placed back in an incubator to continue culture for three weeks until the colonies were formed. A colony is defined as more than 50 cells congregated together. For un-irradiated control, 200 cells were inoculated into a 100-mm Petri dish and allowed to grow. The plating efficiency (PE) was determined, as the percentage of cells seeded to the number that grew into colonies. At least triplicate studies were performed for each time interval for the un-irradiated control group and irradiated group. Surviving fraction (SF) is the ratio of the numbers of colonies produced to the number of cells plated, with a correction necessary for PE, Surviving fraction = colonies counted/[cells seeded × (PE/100)]. The cell survival fraction measurement was repeated three times, and then fitted the survival data to the LQ model.

#### 2.1.2. Doubling Time Study

UMSCC-1 and KB cells from an 80% confluency culture were typsinized, and viable cells were counted through trypan blue staining. 3 × 10^4^ cells per well were plated in a 12-well plate and cultured for 72 h (less than 80% confluency at 72 h). The cells were typsinized, counted, re-plated at a density of 3 × 10^4^ cells per well in a 12-well plate and sub-cultured for another 72 h. The total numbers of cells were recorded to calculate the population doubling time (PDT). The experiment was performed in triplicates each time and was repeated three times. The PDT was calculated using the following equation [11]: 


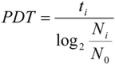
(1)

where *N_i_* is the number of cells at time t_i_, N_0_ is the initial number of cells. The average value of the PDT for each measurement was quoted as the PDT of UMSCC-1 or KB cells, respectively.

### 2.2. Linear-Quadratic Model

The surviving fraction S of cells irradiated to a total dose D within an effective treatment time T is given by Linear-Quadratic formalism (LQ). According to [7,8], the LQ model with repopulation reads as [8]:


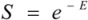
(2)

and:


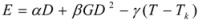
(3)

where the quantity *E* is the biological effectiveness of lethal damage per cell corrected for repopulation effects. *α* and *β* characterize intrinsic radiosensitivity, *G* is the dose protraction factor, which accounts for both dose rate and repair of sub-lethal damage; *γ* is the effective tumor-cell repopulation rate [*γ=ln(2)/T_d_*, *T_d_* is the tumor-cell doubling time], T is overall treatment time, T_K_ is “kick-off” time of accelerated proliferation, meaning the time from the beginning of treatment to the starting of accelerated proliferation. T_k_ for head and neck tumors as revealed by [12] is 28 days, with a range from 21 to 35 days.

The general Lea-Catcheside dose protraction factor *G* is given by: 


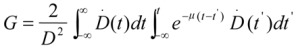
(4)

here, 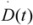 is the dose rate function, *μ* is the repair rate of tumor cells (*μ = ln2/T_γ_*, *T_γ_* is the characteristic repair halftime of cells with sub-lethal damage). For split-dose exposure with two equal fractions, G can be simplified as:


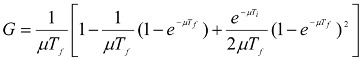
(5)

where, T_i_ is the time interval between the two split fractions, T_f_ is the dose delivery time.

### 2.3. BED, EUD and TCP Models

The radiobiological models, such as biological equivalent dose, equivalent uniform dose, and tumor control probability, were used to measure the treatment effectiveness of different IMRT techniques using the newly derived parameter set.

Biologically effective dose (BED) is the concept used to compare different treatment modalities or fractionation schedules: 


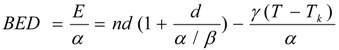
(6)

For conventional EBRT, when the dose-delivering time is much shorter than the repairtime *T_r_* of tumor cells, *D = nd* and *G = 1/n*, where *n* is the number of dose fractions, and *d* is the dose per fraction. γ = ln 2/T_d_ is the effective tumor cell repopulation rate, T_d_ is the potential doubling time. The treatment time *T* of EBRT can be simply calculated as the number of treatment fractions multiplied by 1.4 (5 fractions per week).

The dose inhomogeneities are considered by using the concept of equivalent uniform dose (EUD), which is defined as the dose that, if distributed uniformly, will lead to the same biological effect as the actual non-uniform dose distribution [13]. The EUD for tumor is calculated using the LQ formalism with the parameter set derived from this analysis.

To account for dose heterogeneity, the survival fraction is calculated based on the dose volume histogram (DVH) by: 


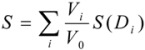
(7)

where V_0_ is the tumor volume, V_i_ is the sub volume corresponding to dose bin D_i_ on the DVH. A representative DVH for a H&N case was used to calculate the EUDs.

The numerical value of EUD is relative to the value delivered by a standard reference regimen, e.g., 2 Gy fraction. By definition, surviving fraction *S* resulting from a dose-delivery regimen was formulated as: 


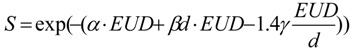
(8)

Thus, the corresponding EUD that results in the surviving fraction *S* can be calculated by: 


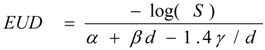
(9)

For a given plan, the surviving fraction *S* is first calculated using the LQ formalism and the above DVHs. Then, the EUD is obtained based on Equation (8).

The tumor control probability (TCP) with clonogen proliferation is also calculated from the cell surviving fraction S shown in Equation (1) using the Poisson hypothesis [14]:


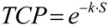
(10)

where, S is the cell surviving fraction shown in Equations (2) and (3), K is the number of tumor clonogens, and is assume to be an arbitrary number (*i.e*., 1 × 10^5^) in the calculation. We also assumed the density of tumor clonogens throughout the tumor was constant.

### 2.4. The Fitting Method

In our study, the least χ^2^ (chi-square) method is used to fit the survival rate from *in vitro* experiment. Three fitting parameters, such as α, α/β, T_r_ are independent variables. The basic idea of this fitting method is that the best-fit curve for a given data set has the minimal sum of the squares of the offsets (the least chi-square error). The sum of the squares of the offsets is defined as:


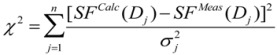
(11)

where *SF*^Meas^(D_j_) is the j-th observed survival rate, *SF*^Calc^(D_j_) is the calculated survival rate for the given dose D_j_; σ^2^(D_j_) is the corresponding statistical error for the j-th data point.

## 3. Results

### 3.1. Repair Halftime T_r_ and Doubling Time Td of HNC

**Figure 1:**
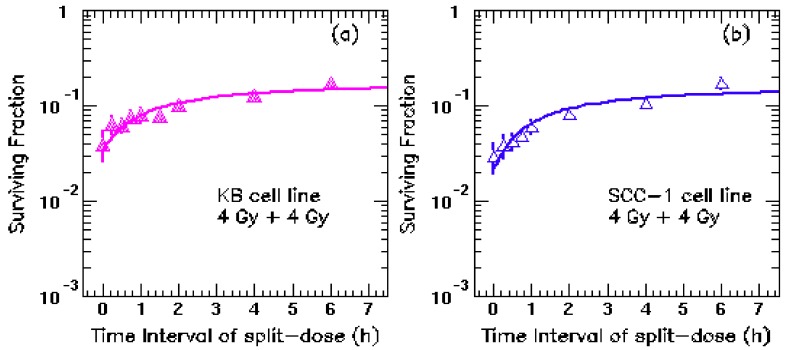
Fitted cell sublethal repair curves using split-doses exposure of 8 Gy. (**a**) KB cell line, the fitting yields: α/β = 8.3 ± 4.1 Gy, α = 0.22 ± 0.08 Gy^−1^ with repairtime T_r_ = 18 ± 21 min; (**b**) the fitted results for UMSCC-1 cell line: α/β = 7.9 ± 5.1 Gy, α = 0.23 ± 0.10 Gy^−1^ with repair halftime T_r_ = 16 ± 25 min. Both cell lines are from HNC cells.

Figure 1 shows the cell surviving fraction as a function of the time interval between split-doses. The split-doses of 4 Gy + 4 Gy were delivered with time intervals of 0, 0.25, 0.5, 0.75, 1.5, 2.0, 4 and 6 h to the two HNC cell lines. The curves are the fitting results based on Equations (2–4) for each cell line separately. For KB cells (shown as Figure 1a), the fitting yields were: α/β = 8.3 ± 4.1 Gy, α = 0.22 ± 0.08 Gy^−1^ with repair halftime T_r_ = 18 ± 21 min; while the fitting results for UMSCC-1 cell line (shown as Figure 1b) were: α/β = 7.9 ± 5.1 Gy, α = 0.23 ± 0.10 Gy^−1^ with repairtime T_r_ = 16 ± 25 min. The derived potential doubling times for UMSCC-1 cell line and KB cell line are T_d_ = 3.6 ± 2.2 days, T_d_ = 4.5 ± 2.8 days, respectively. Similar results were obtained for both cell lines. Combined the results from these two cell lines, we have: α/β = 8.1 ± 4.3 Gy, α = 0.22 ± 0.08 Gy^−1^, the repair halftime T_r_ = 17 ± 21 min and the potential doubling time T_d_ = 4.0 ± 1.8 days. Unless otherwise stated, the parameter set was used in the calculation throughout the paper. Our finding is consistent with previous publications [13,15]. Importantly, the repair halftimes for HNC cells turn out to be very short (~17 min), which may be comparable to the IMRT fraction dose delivery time for step-and-shoot IMRT. Consequently, the cell killing for the HNC IMRT might be affected by the prolonged delivery time.

**Figure 2:**
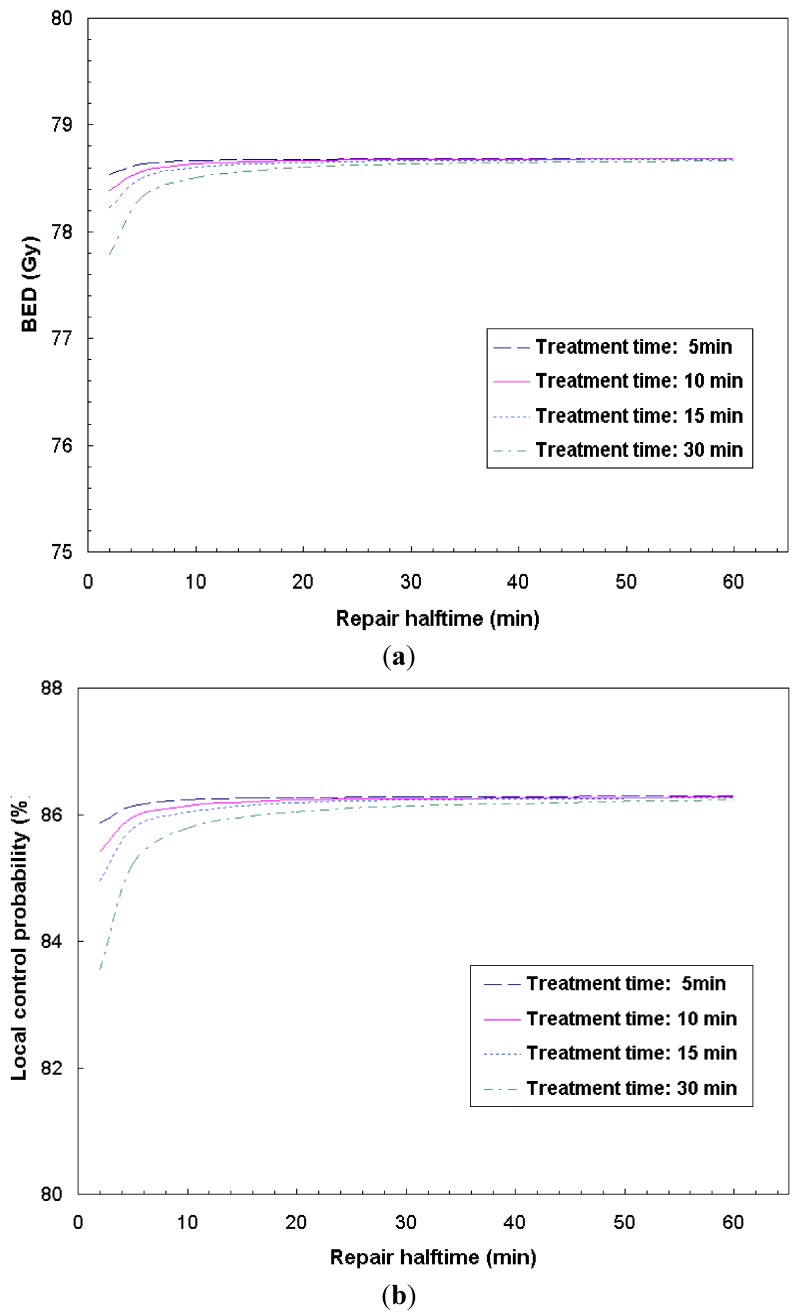
The calculated BED and TCP as a function of repair halftime for H&N tumor. The combined parameter based on the studies of two cell lines from this analysis: α/β = 8.3 ± 4.1 Gy, α = 0.22 ± 0.08 Gy^−1^ and repair halftime T_r_ = 17 ± 21 min, T_d_ = 4.0 ± 1.8 days were used in the calculation. The fraction dose delivery time of 2, 10, 17 and 30 min were calculated and shown.

Impacts of repair halftime on the IMRT treatment effectiveness measured by BED and TCP were computed as a function of delivery times. Figure 2 shows: (a) the calculated BED and (b) TCP as a function of repair halftime. As an example, the fraction dose delivery time of 2, 10, 17 and 30 min was considered. Figure 2a,b indicates longer fraction dose delivery times (*i.e*., the delivery times of 30 min versus 5 min) results in the reduction of BED and TCP, especially for the tumor cells with short repair time. However, it is less a concern for the tumor whose repair halftime is relatively long.

**Figure 3:**
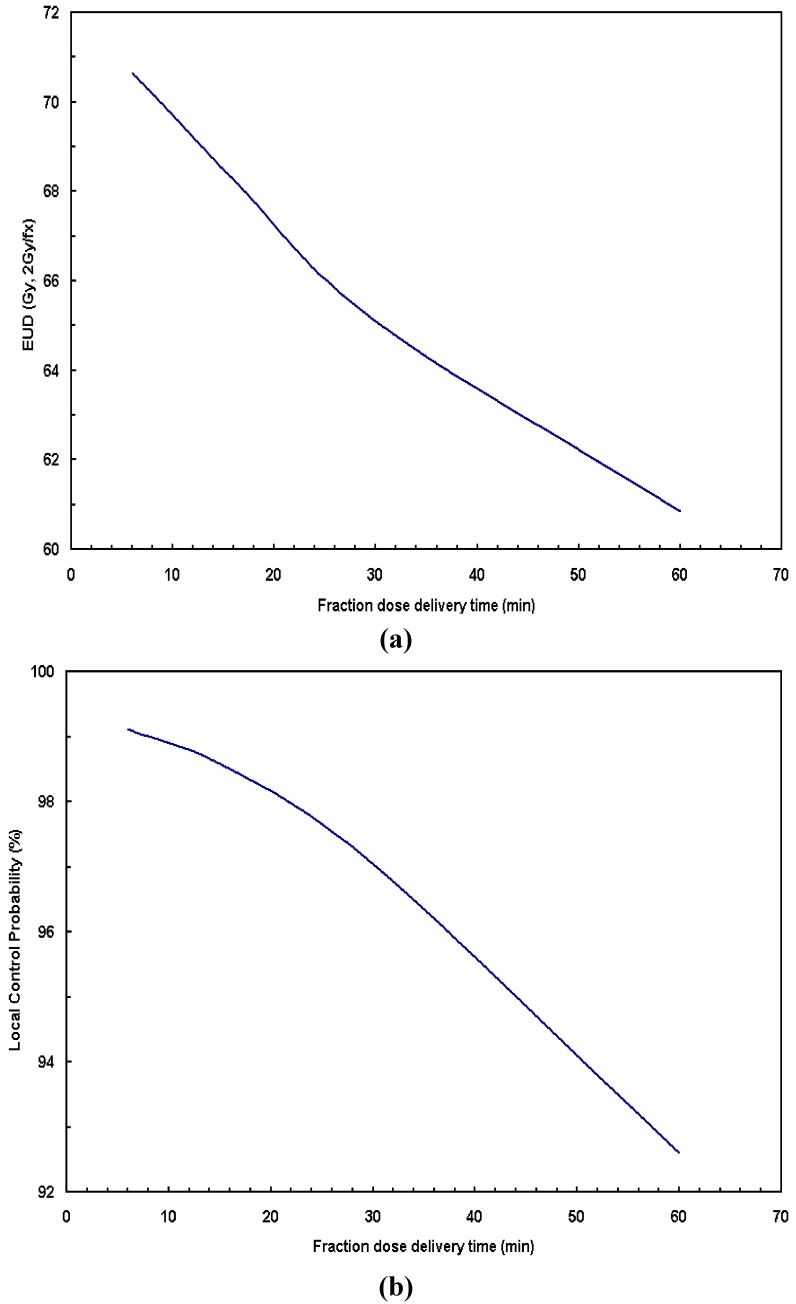
Influences of dose delivery time on (**a**) EUD and (**b**) TCP. The derived parameters: α/β = 8.3 ± 4.1 Gy, α = 0.22 ± 0.08 Gy^−1^ and repair halftime T_r_ = 16 ± 21 min, the potential doubling time T_d_ = 4.0 ± 1.8 days were used in the calculation.

Figure 3 shows (a) EUD and (b) TCP as a function of dose delivery time. Obviously, the prolonged fraction delivery time would result in substantially reduced radiobiological effectiveness for the HNC treatment. For example, a reduction of 6.4% in EUD and 2% in terms of local control is seen by extending the fraction dose delivery time from 10 min to 30 min.

**Figure 4:**
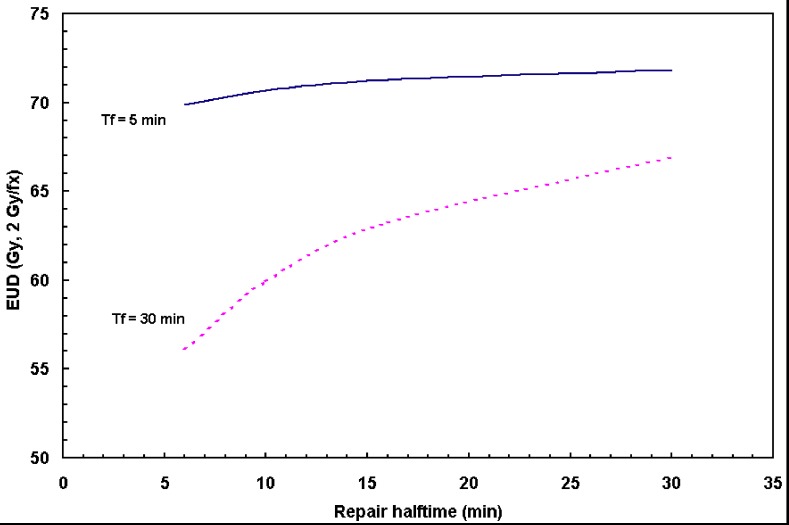
The variation of EUD as a function of repair halftime T_r_. The fraction dose delivery time of 5 min (dashed) and 30 min (solid) were shown. The standard fraction scheme was considered in the calculation: 70 Gy in 2 Gy/fraction.

Figure 4 displays EUD as a function of the repair halftime T_r_, where the dose fraction delivery times T_f_ were assumed to be 5 min (solid) and 30 min (dashed), respectively. Figure 4 reveals that a long fraction dose delivery time T_f_ results in reduced tumor EUD.

Given a repair halftime of 17 min determined from this analysis, for the fraction dose delivery time T_f_ = 5 min, EUD is calculated to be 71.2 Gy; EUD dropped to 63.5 Gy if T_f_ = 30 min was assumed (12% drop compared to that of 5 min dose delivery fraction). Table 1 shows the EUD changes calculated with fraction dose delivery times of 5 min and 30 min, respectively. The upper limit of repair halftime of 38 min was also calculated and tabulated in the same table.

**Table 1:** Comparison of EUD changes assuming different fraction dose delivery time (same DVHs were used). The derived results from this analysis: α/β = 8.3 ± 4.1 Gy, α = 0.22 ± 0.08 Gy^−1^, T_r _= 17 ± 21 min and T_d_ = 4.0 ± 1.8 day were used in the EUD calculation.

	EUD (Gy)	EUD (Gy)	EUD change (%)
	T_f_ = 5 min	T_f_ = 30 min	
T_r_ = 17 min	71.2	63.5	−12.1
T_r_ = 38 min	71.6	67.8	−5.6

### 3.2. Ratio of α/β for HNC

**Figure 5:**
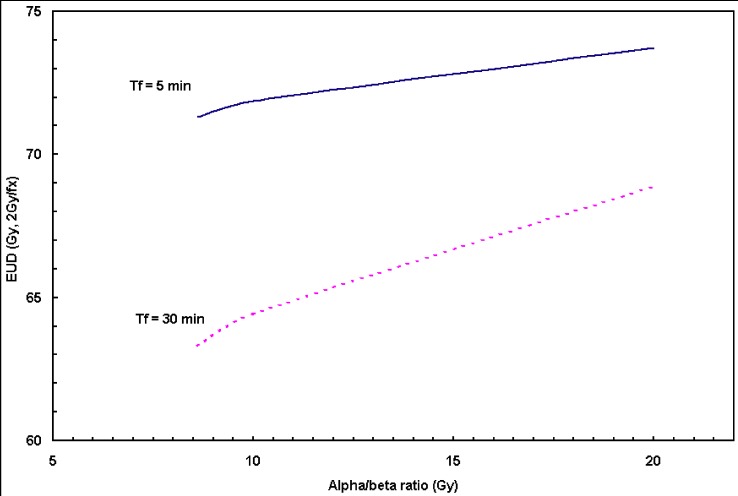
Influence of α/β ratio on EUD for H&N tumor. The result from this analysis α/β = 8.3 Gy was used. The standard fraction scheme was considered in the calculation: 70 Gy in 2 Gy/fraction.

It is generally known that the α/β ratio for H&N carcinomas is great and varies over a large range: 9–20 Gy from the literatures [16,17]. In this analysis, the α/β ratio based on *in vitro* data is found to be 8.3 (range: 8.3–12.4 Gy). A similar value of 8.6 Gy was found from *in vivo* by Fowler *et al*. [16] using RTOG 9003 randomized clinical trial, which is in good accordance with this analysis. Figure 5 shows EUD varies as a function of α/β ratio for HNC assuming dose delivery time of 5 min (solid line) and 30 min (dashed line). A total dose of 70 Gy in 2 Gy fractions was the standard fraction scheme considered in the calculation. Up to 10% of EUD reduction was observed (for T_f_ =30 min) due to the variations of α/β ratios in the range of 8.3–20.0 Gy.

### 3.3. Potential Doubling Time T_d_ of HNC

**Figure 6:**
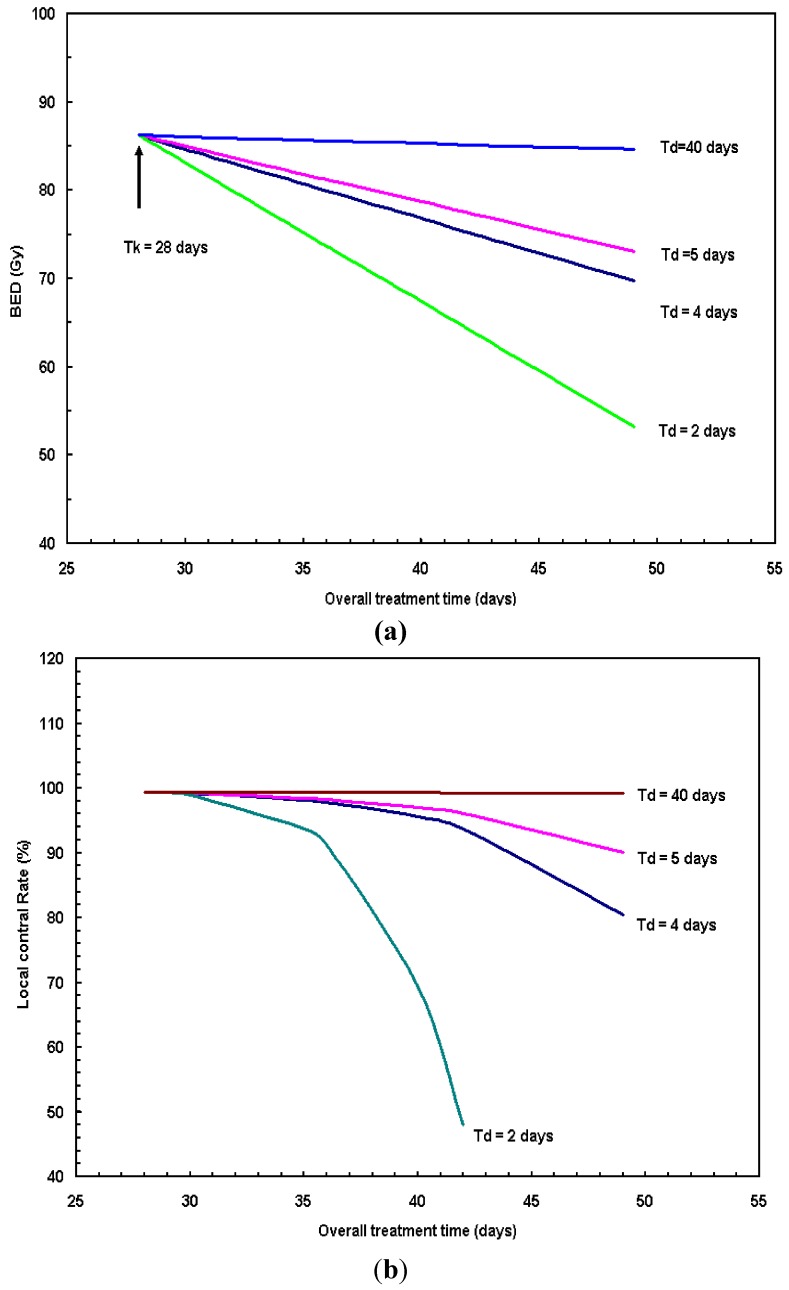
(**a**) Calculated BED and (**b**) local control rate as a function of overall treatment time assuming the potential doubling time T_d_ = 2, 4, 5 and 40 days. The kick-off time T_k_ = 28 day according to [19] was used in the calculation.

Figure 6 presents: (a) the calculated BED and (b) local control rate as a function of overall treatment time assuming the potential doubling time T_d_ = 2, 4, 5 and 40 days. The kick-off time T_k_ = 28 days (range 21–35 days) according to [18] was used in the calculation. For rapidly growing tumors such as HNC, which in general has a short doubling time around 4 days, the overall treatment time was critical in governing tumor control. Assuming the T_d_ = 4 days for HNC, given a standard fraction scheme, a prescription dose of 70 Gy in 2 Gy/fx delivered in 7 weeks, BED was calculated to be 71 Gy; if the overall treatment time was shortened to be within 6 weeks, the BED is 76 Gy, the biological dose gain was then 76–71 = 5 Gy (~6.6% BED increase); the BED gain by shortening overall treatment time from 7 weeks to 6 weeks can go even higher if the doubling time T_d_ is <4 days (larger slope lines shown in Figure 6a). In contrast to HNC, shortening the overall treatment time results in almost no biological advantage for cases of slow proliferating tumors with T_d_ = 40 (or longer) days. The corresponding TCP changes due to the variation of the overall treatment time (T_t_) were shown in Figure 6b, *i.e*., by prolonging T_t_, the TCP drops significantly for HNC when T_r_ < 5 days. Thereby, additional dosing was required to counteract the cell proliferation in the prolonged radiation treatment to maintain the same tumor control rate.

## 4. Discussion

The *in vitro* experiment using two human cell lines of KB and UMSCC-1 was performed to measure the radiosensitivity parameters for HNC. Our analysis indicates relatively rapid repair rate (~17 min) and fast proliferating rate (~4 days) for HNC cells. Such a short repair halftime implies that the prolonged fraction delivery times associated with IMRT may reduce the radiation treatment effectiveness. Other than the dosimetric characteristics, the plan delivery efficiency (due to different IMRT dose delivery techniques) results in different biological effectiveness among different IMRT plans. Currently, a variety of IMRT delivery techniques are available, thus the faction dose delivery times may vary in a large range in order to deliver the same amount of dose. As we have shown in Figure 2, Figure 3, Figure 4, the prolonged dose delivery time generally results in noticeable reduction in BED and EUD, thus a lower tumor control rate. 11% of target EUD reduction was revealed in Figure 4 for the fraction dose delivery time of 30 to 5 min assuming a repair halftime of 17 min for HNC.

Large number of monitor units (MUs) and/or large number of segments, *etc*., may generally result in the prolonged fraction dose delivery time. According to [1], for a Siemens step-and-shoot machine, the estimated fraction dose delivery time can be determined primarily by the number of segments, the number of fields and the total number of MUs per fraction. For a sample H&N case, CMS XiO IMRT plan calls for a total number of segments of 94 (with 8 intensity levels) and a total of 537 monitor units (MUs) for a 7-field IMRT plan. Given a Siemens Primus accelerator with a dose rate of 200 MUs/min, the estimated fraction delivery time is 14.8 min. In general, the number of segments increases with the levels of intensity, for example, the total number of segments is 86, 94, 110 and 117 for the intensity level from 7 to 10, respectively. The estimated fraction dose delivery times are 14.1, 14.8, 16.8 and 17.5 min, respectively. Such fraction dose delivery times are comparable to the sublethal tumor repair halftime of 17 min for HNC, meaning a portion of radiation damaged tumor cells would be repaired during the extended period of dose delivery, resulting in a lower tumor control rate (Figure 3). In order to maintain the same local control, additional doses are needed for IMRT to compensate for the sublethal damage repair. Based on our clinical experience, the total number of segments for a XiO IMRT plan is usually required to be about 100 by adjusting the number of intensity levels. On the other hand, dynamic IMRT generally cuts the dose delivery time to ½ or less, compared to the step-and-shoot technique [2], no appreciable negative treatment effectiveness would be expected in terms of fraction dose deliver time. Some treatment planning algorithm, such as direct aperture optimization (DAO) [20], is able to create plans with smaller number of segments, resulting in shorter fraction delivery time. The novel VMAT technology, generally delivers the desired dose in a single gantry rotation, which may achieve a substantial reduction in treatment delivery time and/or MUs compared to other IMRT approaches [21], resulting in improved treatment effectiveness for HNC treatment.

For fast repopulation cancer cells, it is known that there is a potential decrease in tumor control due to prolonged overall treatment time for radiotherapy of HNC [13,18]. With a doubling time T_d_ estimated to be around 2–4 days, the overall treatment time is an important factor governing tumor control. The optimal fractionation scheme for H&N radiotherapy remains to be determined. As a matter of fact, altered fractionation schedules are being developed and examined to optimize treatment results under various clinical circumstances [7,8,10,17]. For example, in clinical RTOG 9003 randomized trial [10], 1,113 patients were recruited to four radiation fractionation schemes: (1) standard fractionation (SF) at 2 Gy/fx to 70 Gy within 7 weeks; (2) hyperfractionation (HF) at 1.2 Gy/fx BID to 81.6 Gy within 7 weeks; (3) accelerated fractionation with split (AF-S) at 1.6 Gy/fx, BID to 67.2 Gy within 6 weeks; (4) accelerated fractionation with concomitant boost (AF-B) at 1.8 Gy/fx to 72 Gy in 6 weeks. The 2-year local-regional control rates for these 4 arms were 46%, 54.4%, 47.5% and 54.5%, respectively. RTOG 9003 results clearly show that the local control decreases as overall treatment time increases. For example, the 6-week hyperfractionation (HF) scheme has a better local control rate than that of the standard fractionation (SF) scheme (~10% higher in local control rate). Observation from our study (*i.e*., Figure 6a,b) and other literatures [16,17] clearly demonstrate that the longer overall treatment duration without dose escalation has a negative effect on tumor control. Yang *et al*. [22] concluded that significant increase in tumor control can be achieved using accelerated schemes for the tumor with shorter doubling time.

The effectiveness of the linear-quadratic (LQ) model has been demonstrated in both clinical and *in vitro* data. The derived radiobiological parameters based on LQ model from this study (*in vitro*) are in agreement with Fowler *et al*. [16], Mohan *et al*. [17]. However, it should be pointed out that the radiosensitive parameters were derived from *in vitro* cell culture, which could be different from *in vivo* tumor environment in terms of cellular environment and metabolism. Additional *in vivo* experiments are needed to verify the *in vitro* results. Caution needs to be taken to design any clinical trial based on the *in vitro* results.

## 5. Conclusions

Plausible radiobiological parameter sets using two head-and-neck carcinoma cell lines were derived from the present *in vitro* study. The derived parameters (combined results from two HNC cell lines) are: α/β = 8.1 ± 4.3 Gy, α = 0.22 ± 0.08 Gy^−1^, the repair halftime T_r_ = 17 ± 21 min and the potential doubling time T_d_ = 4.0 ± 1.8 day. The estimated α/β ratio from our *in vitro* experiment compared well with the previous results from *in vivo* literatures. The parameter sets derived from *in vitro* suggests that HNC cells have relatively short sub-lethal repair halftimes and rapid proliferating times. As a result, a noticeable reduction in treatment effectiveness can be expected from the prolonged fraction dose delivery times associated with different IMRT delivery techniques. Cautions should be taken in choosing IMRT plan parameters, such as the number of fields and total number of segments, especially for the step-and-shoot IMRT in HNC treatment. The presently derived parameters may be used to design new treatment fractionation schemes and/or to evaluate various IMRT treatment plans. The newly-invented treatment technology (such as VMAT), which provides high quality dose distribution in faster dose delivery fashion, may further improve tumor control in routine clinical use.
